# Multi-omics reveals Dengzhan Shengmai formulation ameliorates cognitive impairments in D-galactose-induced aging mouse model by regulating CXCL12/CXCR4 and gut microbiota

**DOI:** 10.3389/fphar.2023.1175970

**Published:** 2023-04-10

**Authors:** Jing-Yi Hou, He Xu, Guang-Zhao Cao, Liang-Liang Tian, Li-Han Wang, Nai-Qiang Zhu, Jing-Jing Zhang, Hong-Jun Yang

**Affiliations:** ^1^ Beijing Key Laboratory of Traditional Chinese Medicine Basic Research on Prevention and Treatment for Major Diseases, Experimental Research Center, China Academy of Chinese Medical Sciences, Beijing, China; ^2^ Robot Intelligent Laboratory of Traditional Chinese Medicine, Experimental Research Center, China Academy of Chinese Medical Sciences and MEGAROBO, Beijing, China; ^3^ Postdoctoral Mobile Research Station of China Academy of Chinese Medicine Sciences, Beijing, China; ^4^ Institute of Chinese Materia Medica, China Academy of Chinese Medical Sciences, Beijing, China

**Keywords:** Dengzhan Shengmai (DZSM), cognitive impairment (CI), aging, CXCR4, CXCL12

## Abstract

Dengzhan Shengmai (DZSM), a traditional Chinese medicine formulation, has been administered extensively to elderly individuals with cognitive impairment (CI). However, the underlying mechanisms by which Dengzhan Shengmai improves cognitive impairment remains unknown. This study aimed to elucidate the underlying mechanism of the effect of Dengzhan Shengmai on aging-associated cognitive impairment *via* a comprehensive combination of transcriptomics and microbiota assessment. Dengzhan Shengmai was orally administered to a D-galactose-induced aging mouse model, and evaluation with an open field task (OFT), Morris water maze (MWM), and histopathological staining was performed. Transcriptomics and 16S rDNA sequencing were applied to elucidate the mechanism of Dengzhan Shengmai in alleviating cognitive deficits, and enzyme-linked immunosorbent assay (ELISA), quantitative real-time polymerase chain reaction (PCR), and immunofluorescence were employed to verify the results. The results first confirmed the therapeutic effects of Dengzhan Shengmai against cognitive defects; specifically, Dengzhan Shengmai improved learning and impairment, suppressed neuro loss, and increased Nissl body morphology repair. Comprehensive integrated transcriptomics and microbiota analysis indicated that chemokine CXC motif receptor 4 (CXCR4) and its ligand CXC chemokine ligand 12 (CXCL12) were targets for improving cognitive impairments with Dengzhan Shengmai and also indirectly suppressed the intestinal flora composition. Furthermore, *in vivo* results confirmed that Dengzhan Shengmai suppressed the expression of CXC motif receptor 4, CXC chemokine ligand 12, and inflammatory cytokines. This suggested that Dengzhan Shengmai inhibited CXC chemokine ligand 12/CXC motif receptor 4 expression and modulated intestinal microbiome composition by influencing inflammatory factors. Thus, Dengzhan Shengmai improves aging-related cognitive impairment effects *via* decreased CXC chemokine ligand 12/CXC motif receptor 4 and inflammatory factor modulation to improve gut microbiota composition.

## Highlights

• DZSM could improve cognitive impairments in elderly individuals.
• Multi-omics indicated that CXCR4 and its ligand (CXCL12) are the targets for improving cognitive impairment effects by DZSM, and also indirectly modulated the intestinal flora composition (*Bacteroidia*, *Erysipelotrichia*, and *Deferribacteres*).
• *In vivo*, enzyme-linked immunosorbent assay, quantitative real-time PCR, and immunofluorescence further confirmed that DZSM suppressed expression of CXCR4, CXCL12, and inflammatory cytokines (IL-18, IL-1β, IL-6, and TNF-α).

## Introduction

Aging is naturally driven, regulated by multiple hormonal signaling pathways, and influenced by the interactions of various genetic and environmental factors (Estebsari et al., 2020). According to the World Population Prospects report, the percentage of the aging population of the world is expected to reach 16% by 2050 with improvements in the healthcare system, living conditions, and social welfare (Li et al., 2022). With global aging, the incidence of cognitive impairment (CI) continues to increase each year. CI refers to the gradual loss of cognitive ability and learning and memory ability (Eshkoor et al., 2015), seriously affecting quality of life and causing a heavy burden on families and society (Pei et al., 2020). Currently, the number of drugs for the treatment of age-related CI is very limited. These drugs include cholinesterase inhibitors (Battle et al., 2021) and excitatory amino acid receptor antagonists (Wang and Huang, 2018), but they have not been included in the standardized clinical treatment plans, as they have failed to show clinical treatment benefits (O'Brien et al., 2017). Therefore, the development of new therapies and drugs for improving CI and promoting the recovery of cognitive function in elderly adults still faces considerable challenges.

Dengzhan Shengmai (DZSM), a formulation derived from “Shengmai San,” primarily contains four traditional Chinese medicine (TCM) ingredients, namely, the medicinal part of *Erigeron breviscapus* (Vaniot) Hand.-Mazz. (rhizoma), the medicinal part of *Panax ginseng* C.A. Mey., (rhizoma), the medicinal part of *Ophiopogon japonicas* (Thunb.) Ker-Gawl. (tubers), and the medicinal part of *Schisandra chinensis* (Turcz.) Baill., (dried fruit), in a weight ratio of 5:1:1:1.8 (drug approval number in Chinese Food and Drug Administration: Z20026439) (Mu et al., 2019). Among these TCM ingredients, *P. ginseng* C.A. Mey., and *S. chinensis* (Turcz.) Baill., the noted adaptogenic herbs (Slanina et al., 1997; Ratan et al., 2021), have been utilized as official medicine in Russia, as well as recorded in Chinese Pharmacopoeia (2020 edition) (Chinese Pharmacopoeia Commission, 2020), Russian Federation’s State Pharmacopoeia (14th edition) (The State Pharmacopoeia of Russian Federation, 2018), European Pharmacopoeia (10 edition) (European Pharmacopoeia, 2019), Japanese Pharmacopoeia (18 edition) (Society of Japanese Pharmacopoeia, 2021), and American herbal pharmacopoeia (2021 edition) (American herbal pharmacopoeia, 2021), and are known to have the following effects: nourishing blood, promoting blood circulation, invigorating vital energy, and nourishing *Qi*. *Ophiopogon* (Thunb.) Ker-Gawl., nourishes *Yin*, and is known to promote circulation and cleanse the heart. *S. chinensis* (Turcz.) Baill., functions as an astringent and nourishes *Qi*. *E. breviscapus* (Vaniot) Hand.-Mazz., promotes blood circulation and dredges collaterals. DZSM has been used clinically to improve cognitive dysfunction and forgetfulness for many years, and its efficacy and safety have also been reported in many clinical and experimental studies. Song et al., found that long-term use of DZSM could improve the score of Mini-mental State Examination (MMSE), Montreal Cognitive Assessment (MoCA), and Barthel Index, suggesting that DZSM effectively improves CIs and cognitive dysfunctions (Song et al., 2018). A meta-analysis of randomized clinical trials also indicated that DZSM appears to alleviate neurological function impairment and improves quality of life in elderly individuals. In addition, Sheng et al. (2017) and Zhang et al. (2020) demonstrated that DZSM and its active components effectively protect against cognitive deficits of Alzheimer’s Disease (AD) and improve aging-related learning and memory deficits by accelerating the aggregation of A*β*, reducing A*β*-oligomer formation, and mediating changes in white matter microstructure functions. As the underlying mechanism of multi-ingredient and multi-pharmacological formulations to improve aging-related neurocognitive impairment has not yet been fully elucidated, further research is needed.

Owing to the rise of “omics” technologies, the underlying mechanisms of TCM in the treatment of complex and chronic diseases have been systematically elucidated in multiple dimensions and levels (Hou et al., 2022). Among these “omics” technologies, transcriptomics is based on the study of overall gene transcription and transcriptional regulation (Hou et al., 2021), while the study of intestinal flora is based on the distribution and composition of gut microbiotas (Lin et al., 2021). The systematic integration of multi-omics provides a systematic mechanistic understanding of multi-component formulas targeting complex diseases through multi-targeting (Yang et al., 2022). In this study, we investigated the role of DZSM against aging-associated CIs by further exploring how DZSM ameliorates CI in a D-galactose-induced aging mouse model based on the comprehensive integration of transcriptomics and gut microbiota, attempting to provide a scientific basis for transcriptomic microbiome-based intervention in CI.

## Materials and methods

### Chemicals and reagents

DZSM formulation dry powder voucher specimens were provided by Yunnan Bio Valley (China, No: 20190023). D-galactose (purity, ≥99%) and melatonin (purity, ≥99%) were purchased from Sigma-Aldrich (St. Louis, United States). A CXC chemokine ligand 12 (CXCL12)-targeting antibody and the chemokine CXC motif receptor 4 (CXCR4)-targeting antibody were purchased from Proteintech Group Co., Ltd., (Chicago, IL). DNase/RNase free water, phosphate-buffered saline (PBS) and Bovine Serum Albumin (BSA) were purchased from Solarbio (Beijing, China). A FastPure RNA Extraction Kit and SYBR qPCR Master Mix were purchased from Vazyme Biotech Co., Ltd., (Nanjing, China). An M5 Super Plus qPCR RT kit was purchased from Mei5 Biotechnology Co., Ltd., (Beijing, China). Enzyme-linked immunosorbent assay (ELISA) kits for CXCL12, CXCR4, interleukin-1β (IL-1β), interleukin-6 (IL-6), interleukin-18 (IL-18), and tumor necrosis factor-α (TNF-α) were purchased from Elabscience Biotechnology Co., Ltd., (Wuhan, China).

### UPLC/MS for chemical fingerprints

The DZSM formulation dry powder was accurately weighted (0.50 g) and ultrasonically extracted with 40 mL of 50% methanol for 60 min; the supernatant was then filtrated before analysis, using a 0.45 μm filter. A HPLC system coupled with an Orbitrap mass spectrometer (Agilent, United States) was applied for the LC-MS analysis, and a Leapsil (C18, 2.7 μm, 150 mm × 2.1 mm) was used for all analyses. The mobile phase comprised of 0.1% formic acid-water (A) and 0.1% formic acid-acetonitrile (B). These were used for the gradient elution program as follows: 0%–15% B (0–1 min); 15%–30% B (1–29 min); 30%–32% B (29–31 min); 32%–38% B (31–37 min); 38%–43% B (37–48 min); 43%–55% B (48–54 min); 55%–70% B (54–70 min); 70%–90% B (70–76 min); and 90% B (76–86 min). We loaded 2 μL of the samples with a flow rate of 300 μL/min for the mobile phase. In the positive/negative polarity mode, electrospray ionization (ESI) source was employed with a scanning model of Spray Voltage (kV) at 3.6 and 3.3 kV, respectively. Full MS/dd-MS^2^ at full scan (m/z 100–1,700) was performed while maintaining the capillary temperature at 350°C. Subsequently, Compound Discoverer 3.2 was utilized for retention time correction and peak extraction, and mzCloud online database (https://www.mzcloud.org/) was employed for substance identification based on secondary spectrum information.

### Animal grouping and medication treatment

**FIGURE 1:**
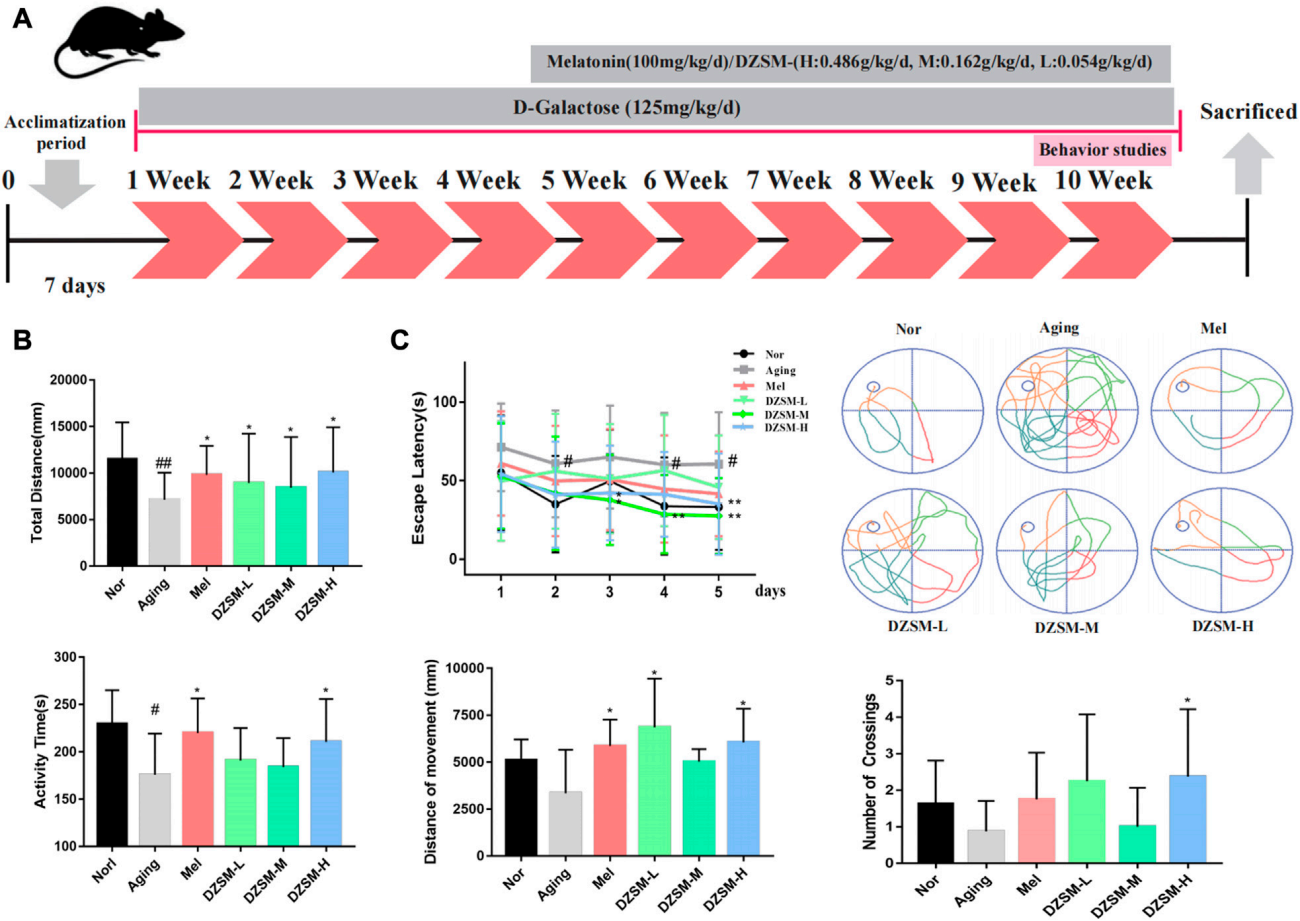
Experimental design and behavioral analysis. **(A)** Experimental design for medication treatment in C57BL/6 mice and behavioral analysis; **(B)** Open field task: a. total movement distance, b. activity time; **(C)** Morris water maze: a. latency to platform in the 5-day training phase, b. movement tracking in the positioning navigation phase, c. movement distance in target quadrant, d. number of platform crossings. (Compared with the Nor group: ^#^ *p* < 0.05, ^##^ *p* < 0.01; compared with the Aging group: ^*^ *p* < 0.05, ^**^ *p* < 0.01, *n* = 8).

Male C57BL/6J mice (age, 16 weeks; weight, 25–28 g) were purchased from Beijing Jinmuyang Biotechnology Co., Ltd., (Beijing, China; certification No. SCXK (Jing) 2019-0010). All experimental procedures were performed in accordance with the rules of care and the use of basic theories of Chinese Medical Sciences (Approval No. 2022B029). All animals were kept at 25.0ºC ± 1.0°C with a 12-h light-dark cycle and free access to food and water. As a neuroendocrine hormone, melatonin exhibits a wide range of physiological activities, including reducing oxidative stress, immune modulation, neuroprotection, improving CI, and improving learning and memory (Dai et al., 2021). Therefore, we selected melatonin as the positive drug to control in the current study. After 7 days of adaptation, the mice were randomly divided into six groups (*n* = 10): normal (Nor), aging (Aging), melatonin (Mel), and DZSM (DZSM-L, DZSM-M, and DZSM-H) groups. As shown in Figure 1A, the CI model was established by intraperitoneal injection of D-galactose (125 mg/kg/d) for 10 weeks. Mice in the Mel and DZSM groups (DZSM-L, DZSM-M, and DZSM-H) received oral melatonin (100 mM/kg) or DZSM (0.054 g/kg/d, 0.162 g/kg/d, 0.486 g/kg/d), respectively, for 6 weeks, after 4 weeks of D-galactose induction. Behavioral testing was carried out during the 10th week, after which, all mice were euthanized humanely, and their brain tissues were collected.

### Behavioral observations

#### Open field task (OFT)

An OFT was used to evaluate the locomotor activity of the mice. Briefly, the mice were independently placed within an empty area (30 cm × 30 cm) and permitted to move freely for 3 min to obtain the data.

#### Morris water maze (MWM)

A MWM experiment was performed to detect escape latency and spatial positioning. The experimental procedure was carried out for 7 days. On the first day, the mice were placed in the pool to adapt to the environment. Each mouse was randomly left to find the platform within 1 min on days 2–5. If the mice reached the platform within the allotted time, they were allowed to rest at the platform for 15 s; mice failing to reach the platform within 1 min were directed to the platform and allowed to rest for 15 s. On day 6, the platform was hidden below the surface, and the escape time of each mouse was tested for 60 s. On the last day, after the removing the platform, we recorded the frequency with which each mouse crossed the location where the platform had previously been located.

### Histopathological staining

The harvested brain tissues were fixed with 4% paraformaldehyde for 24 h, and the fixed brain slices were dehydrated and embedded to prepare paraffin sections. The paraffin samples were cut into 5-μm sections for staining with hematoxylin and eosin (H&E) and Nissl, after which, the histopathological changes of the brain tissues could be examined.

### Transcriptomics analysis using high-throughput sequencing

Total RNA was extracted from the brain tissues of mice in the Nor, CI, and DZSM-H groups (*n* = 3) using TRIzol Reagent. Applied Protein Technology (Shanghai, China) constructed the cDNA libraries using RNA, and they were sequenced on an Illumina Novaseq 6000/MGISEQ-T7 instrument according to a standardized sequencing protocol. Differentially expressed genes (DEGs) between groups were analyzed using the “limma” package and determined according to the conditions as follows: |log2(Fold Change)| > 0.58 and *p*-value < 0.05. Gene Ontology (GO) and Kyoto Encyclopedia of Genes and Genomes (KEGG) enrichment of DEGs at both Aging-vs.-Nor and DZSM-H-vs.-Aging groups were implemented by the DAVID database (https://david.ncifcrf.gov/) and Metascape (https://metascape.org/gp/index.html#/main/step1). The protein-protein network (PPI) of reversed DEGs was constructed by STRING database (https://cn.string-db.org/), and visualized in Cytoscape 3.7.2 platform. The topological parameter of nodes and interaction edges in the network was calculated by cytoHubba plug-in, and the nodes with top degrees were considered as key targets.

### 16S rRNA high-throughput sequencing for gut microbiota

Ten mice were randomly selected from the Nor, CI, and DZSM-H groups, and 0.1 g of fresh feces was collected. Microbial DNA was first extracted using the HiPure Soil DNA Kits. Following the extraction of microbial DNA, the V3+V4 region of 16S rDNA was amplified with specific barcoded primers. The PCR product was quantified by the ABI StepOnePlus Real-Time PCR System (Life Technologies, United States). The samples were sequenced on an Illumina platform by Genedenovo Biotechnology Co., Ltd., (Guangzhou, China). After sequencing the raw reads, UPARSE was applied to cluster tag (>97%) identities into operational taxonomic units (OTUs); the number of OTUs were then computed. The composition of microbiota, α-diversity, and β-diversity analyses were performed. Thereafter, linear discriminant analysis (LDA) effect size (LEfSe), with a threshold size of >2, was applied to compare differences in the taxa between the Aging-vs.-Nor and DZSM-vs.-Aging groups. Redundancy analysis (RDA) and Spearman analysis were employed to identify the correlations between key targets/inflammatory factors and microorganisms.

### Enzyme-linked immunosorbent assay (ELISA)

Harvested brain tissues (*n* = 8–10) were homogenized in PBS with a glass homogenizer on ice, and the protein concentrations were quantified using a BCA Protein Assay Kit. The levels of CXCL12, CXCR4, IL-1β, IL-6, IL-18, and TNF-α in the brain tissue were determined using the ELISA kits, according to the following procedures: the samples were added to the wells and immobilized with specific antibodies for 90 min; they were incubated with biotinylated detection Ab working solution for 60 min; and the wells were triple washed with washing buffer. Corresponding horseradish peroxidase (HRP) conjugate working solution was then added for another 30 min of incubation, followed by 5-fold washing with a washing buffer before measurements.

### Quantitative real-time PCR

The FastPure RNA Extraction Kit was applied to the extracted total RNA from brain tissues (*n* = 3), which reverse-transcribed to cDNA using the M5 Super Plus qPCR RT kit. The SYBR qPCR Master Mix was then applied to detect mRNA expression. Primers for the detected genes are listed in Supplementary Table S1. The relative expression of mRNA was calculated as 2^−ΔΔCT^.

### Immunofluorescence (IF) staining

Briefly, the brain tissue sections (*n* = 3) were permeabilized with Triton X-100, followed by 5% BSA for sealing, and incubated with anti-CXCR4 (1:150 with 2% BSA) and anti-CXCL12 (1:100 with 2% BSA) antibodies overnight at 4°C. On the following day, the corresponding secondary antibodies (diluted 1:200) were incubated on the sections with DAPI (1:1000). Finally, images of the sections were captured under a fluorescence microscope.

### Statistical analysis

Statistical analyses were performed using GraphPad Prism 7.0 software, and all data analyses were conducted with one-way variance analysis (ANOVA) and the Tukey *post-hoc* test. A *p*-value of <0.05 was considered significant.

## Results

### Identification of chemical components of DZSM

As shown in Table 1, a preliminary analysis revealed 21 ingredients detected by UPLC-MS/MS, including 4-CDOA, Eriodictyol, 4,5-diCQA, Erigoster B, Narigenin-7-glucuronide, Breviscapine, Luteolin, Ginsenoside f1, Ginsenoside Rg1, Ginsenoside Rk1, Ginsenoside Rh4, Schisandrin, Schisandol A, gomisin o, gomisin f, Schisandrin A, 4-CQA, Narigenin-7- glucuronide, acetyl-di-CQA, Notoginsenoside L, and Ginsenoside Re. The total ion chromatogram (TIC) is shown in Supplementary Figure S1.

**TABLE 1:** Results of UPLC-MS/MS analysis of DZSM.

NO.	RT time (min)	Molecular weight	Formula	Mode	Ingredients
1	6.68/4.393	382.08	C_17_H_18_O_10_	Positive/Negative	4-CDOA
2	8.33	288.06	C_15_H_12_O_6_	Positive	Eriodictyol
3	10.85/17.65	516.12	C_25_H_24_O_12_	Positive/Negative	4,5-diCQA
4	12.94/13.60	544.12	C_26_H_24_O_13_	Positive/Negative	Erigoster B
5	13.35	448.10	C_21_H_20_O_11_	Positive	Narigenin-7-glucuronide
6	13.96/14.36	446.08	C_21_H_18_O_11_	Positive/Negative	Breviscapine
7	16.75/10.60	286.04	C_15_H_10_O_6_	Positive/Negative	Luteolin
8	18.49/36.43	638.44	C_36_H_62_O_9_	Positive/Negative	Ginsenoside f1
9	33.12/33.55	800.49	C_42_H_72_O_14_	Positive/Negative	Ginsenoside Rg1
10	36.43/50.064	766.48	C_42_H_70_O_12_	Positive/Negative	Ginsenoside Rk1
11	37.34/54.13	620.42	C_36_H_60_O_8_	Positive/Negative	Ginsenoside Rh4
12	38.01	400.18	C_23_H_28_O_6_	Positive	Schisandrin
13	45.63	432.21	C_24_H_32_O_7_	Positive	Schisandol A
14	51.07	416.18	C_23_H_28_O_7_	Positive	gomisin o
15	54.31	514.22	C_28_H_34_O_9_	Positive	gomisin f
16	66.92	416.21	C_24_H_32_O_6_	Positive	Schisandrin A
17	3.83	354.09	C_16_H_18_O_9_	Negative	Narigenin-7-glucuronide
18	7.04	448.09	C_21_H_20_O_11_	Negative	Narigenin-7-glucuronide
19	24.95	558.13	C_27_H_26_O_13_	Negative	acetyl-di-CQA
20	38.06	1078.59	C_53_H_90_O_22_	Negative	Notoginsenoside L
21	41.79	946.54	C_48_H_82_O_18_	Negative	Ginsenoside Re

### DZSM attenuated cognitive deficits and pathological changes

The OFT and MWM tests were performed at week 10 to assess changes in autonomous behavior, exploration and learning, and memory abilities. As shown in Figure 1B, compared with those in the Nor group, the total distance and activity time were significantly reduced in the Aging group. Compared with those of the Aging group, the movement distance was significantly larger in all medication groups, activity times were somewhat increased in the DZSM-L and DZSM-M groups, and activity times were significantly increased in the DZSM-H and Mel groups. In Figure 1C, the MWM results showed that in the positioning and navigation experiment, compared with that of the Nor group, the initiation period of the Aging group was significantly prolonged; the initiation periods of all medication groups were significantly shorter, especially those of the DZSM-M group on days 3–5, which were significantly reduced. In the space exploration experiment, compared with measurements in the Nor group, the movement distances of the Aging group decreased in the target quadrant, and the number of platform crossings decreased. In contrast, the movement distances within the target quadrant and the number of platform crossings in the medication groups increased significantly. Movement distances in the target quadrant were significantly increased in the Mel, DZSM-L, and DZSM-H groups, and the number of platform crossings was significantly increased in the DZSM-H group. These results provide evidence that DZSM was beneficial for improved learning and memory ability and recovery of cognitive performance in D-galactose-induced aging mice.

**FIGURE 2:**
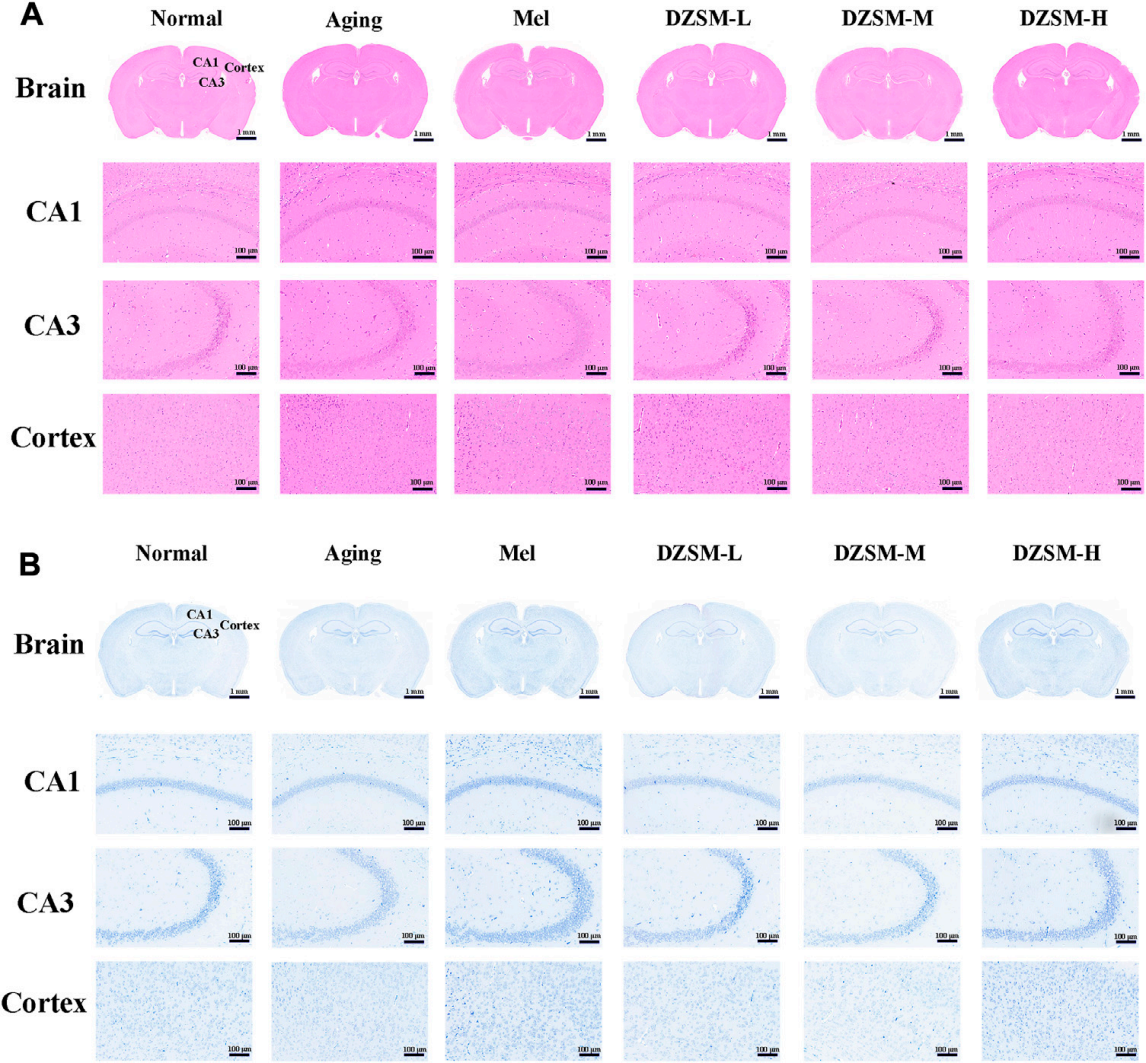
Histopathological changes in the brain tissue. **(A)** H&E staining; **(B)** Nissl staining (*n* = 3). Bar length: 1 mm, 100 μm.

As shown in Figure 2A, H&E staining showed that compared with those in the Nor group, neurons in the Aging group were sparsely arranged, the number of irregularly shaped cells increased, and cell nuclei shrank. Significantly less neuron reduction was observed in mice treated with DZSM. In Figure 2B, on Nissl staining, no obvious pathological changes were discovered in cells of the Nor group, while the number of Nissl bodies was significantly reduced, and neuronal morphology was disrupted in the D-galactose-induced Aging group. The number of Nissl bodies was increased, and their morphologies were better in the Mel and DZSM groups (DZSM-L, DZSM-M, DZSM-H), suggesting that DZSM might effectively reduce D-galactose-induced brain tissue damage in aging mice.

### Identification of targets for the amelioration of CI by DZSM using transcriptomics analysis

**FIGURE 3:**
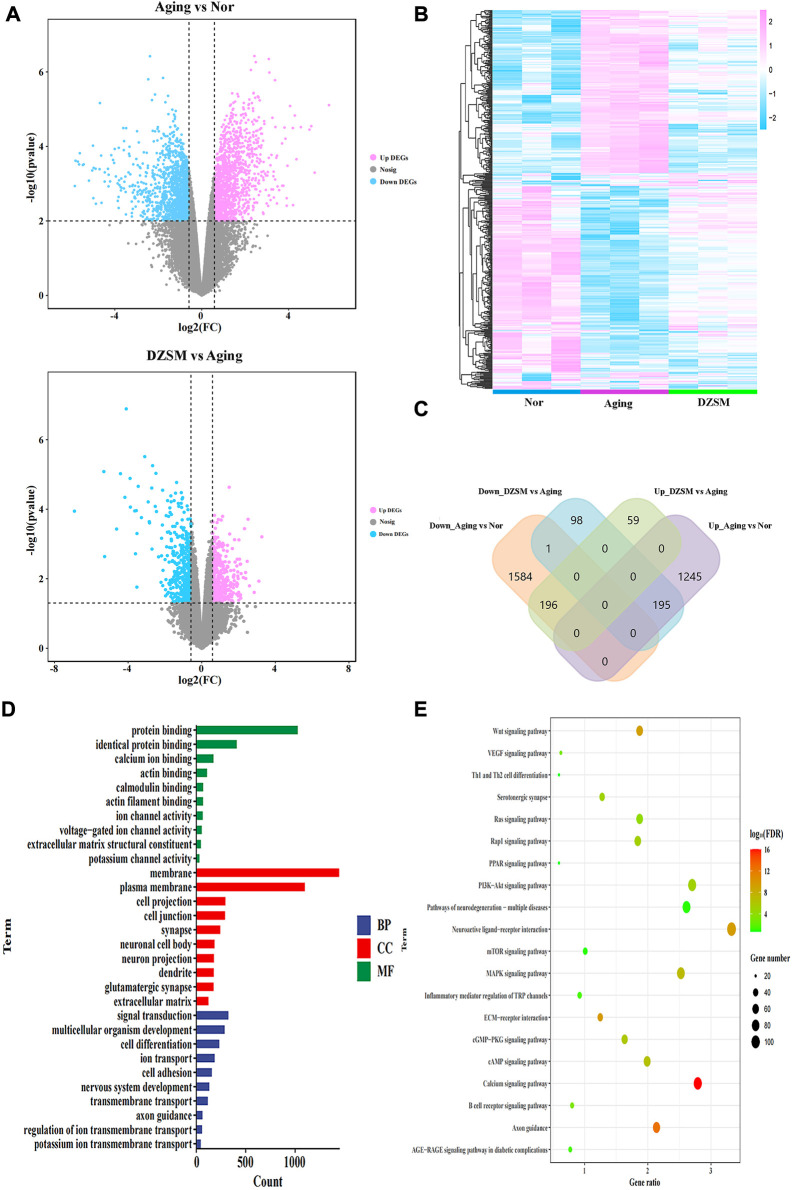
Transcriptomics analysis. **(A)** Volcano map of the DEGs in the Aging vs. Normal (Nor) groups and the DZSM vs. Aging groups; **(B)** Heatmap of the DEGs in the Nor, Aging, and DZSM groups; **(C)** Venn diagram of the DEGs in the Nor, Aging, and DZSM groups; **(D)** GO enrichment analysis of DEGs (red terms represent MF, green terms represent CC, and blue terms represent BP). **(E)** KEGG pathway analysis of DEGs.

The DEGs were screened based on significant *p*-values < 0.05 and |log2(Fold Change)| > 0.58 (Figure 3A). Measured against the Nor group, 3220 DEGs were identified in the Aging group, with 1,440 of them upregulated and 1,780 downregulated. After DZSM treatment, 548 DEGs were identified, with 255 upregulated and 293 downregulated. As shown in Figures 3B, C, the results of hierarchical cluster analysis suggested that after DZSM administration, some gene expression induced by D-galactose was reversed. Venn mapping was used to further clarify the DEG distribution after DZSM administration. These results suggested that D-galactose upregulated the expression of 1,440 DEGs. DZSM reversed the upregulation of 195 DEGs present in the Aging-vs.-Nor groups and downregulation of DEGs in the DZSM-vs.-Aging groups. By comparing the downregulated and upregulated genes in the Aging-vs.-Nor and DZSM-vs.-Aging groups, respectively, we also observed that the expression of 1,781 DEGs was downregulated after D-galactose treatment. The expression of 196 of these DEGs was reversed after DZSM administration, indicating that DZSM might ameliorate the aging effects induced by D-galactose. Enrichment analysis of the DEGs in the Aging-vs.-Nor groups and the DZSM-vs.-Aging groups was performed to elucidate the origins of DZSM amelioration of cognitive deficits in aging mice. As shown in Figure 3D, biological process (BP) analysis indicated that the enriched DEGs in the Aging-vs.-Nor groups and the DZSM-vs.-Aging groups were closely associated with signal transduction. The cellular component (CC) terms were mainly related to membrane and extracellular matrix, and the molecular function (MF) terms were related to the protein and identical protein binding. Furthermore, pathway analysis has suggested that the DEGs were enriched mainly in inflammation and signal transduction, such as the “Th1 and Th2 cell differentiation,” “Wnt signaling,” “Ras signaling,” and “MAPK signaling” pathways (Figure 3E).

**FIGURE 4:**
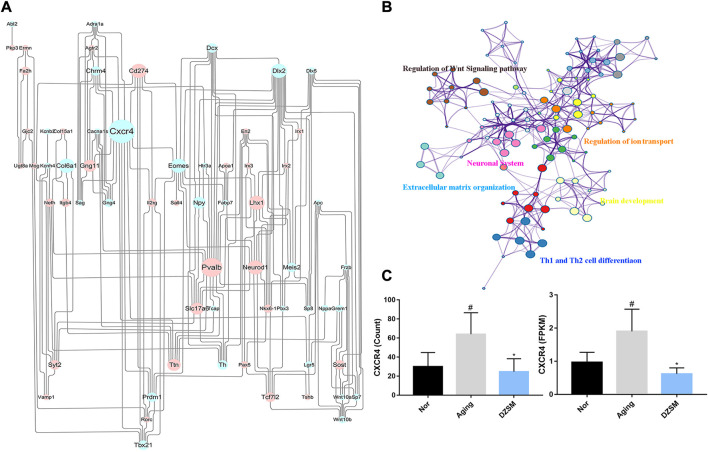
Network analysis of reverse DEGs. **(A)** The network of DEGs in the Aging and DZSM groups. Upregulated/downregulated DEGs are indicated by the blue/pink nodes whose size reflects the degree. **(B)** GO terms for the network of reverse DEGs. **(C)** The expression levels of CXCR4 in RNA-seq (compared with the Nor group: ^#^ *p* < 0.05, ^##^ *p* < 0.01; compared with the Aging group: ^*^ *p* < 0.05, ^**^ *p* < 0.01).

To further explore the potential mechanism of DZSM in attenuating cognitive deficits in aging mice, the identified upregulated/downregulated DEGs in the Aging-vs.-Nor groups and the DZSM-vs.-Aging groups were uploaded to the STRING online database (https://string-db.org/) (combined score of >0.7). A PPI network was then constructed, and the Cytoscape 3.7.2 platform was used to visualize the PPI network with 251 nodes and 506 interaction edges (Figure 4A). Based on the network’s topological properties calculated using the cytoHubba plug-in, CXCR4 (degree = 19) emerged as the principal target in the PPI network, indicating that it may be the key target affected by DZSM. Furthermore, the DEGs with reversed expression between the Aging-vs.-Nor and DZSM-vs.-Aging groups were enriched mainly in GO terms associated with inflammation and signal transduction, including “regulation of the Wnt signaling pathway,” “regulation of ion transport,” “Th1 and Th2 cell differentiation,” “neuronal system,” “extracellular matrix organization,” and “brain development” (Figure 4B). The results of RNA-seq also demonstrated that DZSM significantly reduced the expression levels of CXCR4 and CXCL12 in the Aging group at the FPKM and count levels (Figure 4C). Given these results, we preliminarily speculate that DZSM may regulate the processes of inflammation and signal transduction to ameliorate cognitive function in D-galactose–induced aging mice.

### DZSM reduced expression of CXCL12/CXCR4 and inhibited expression of pro-inflammatory cytokines

**FIGURE 5:**
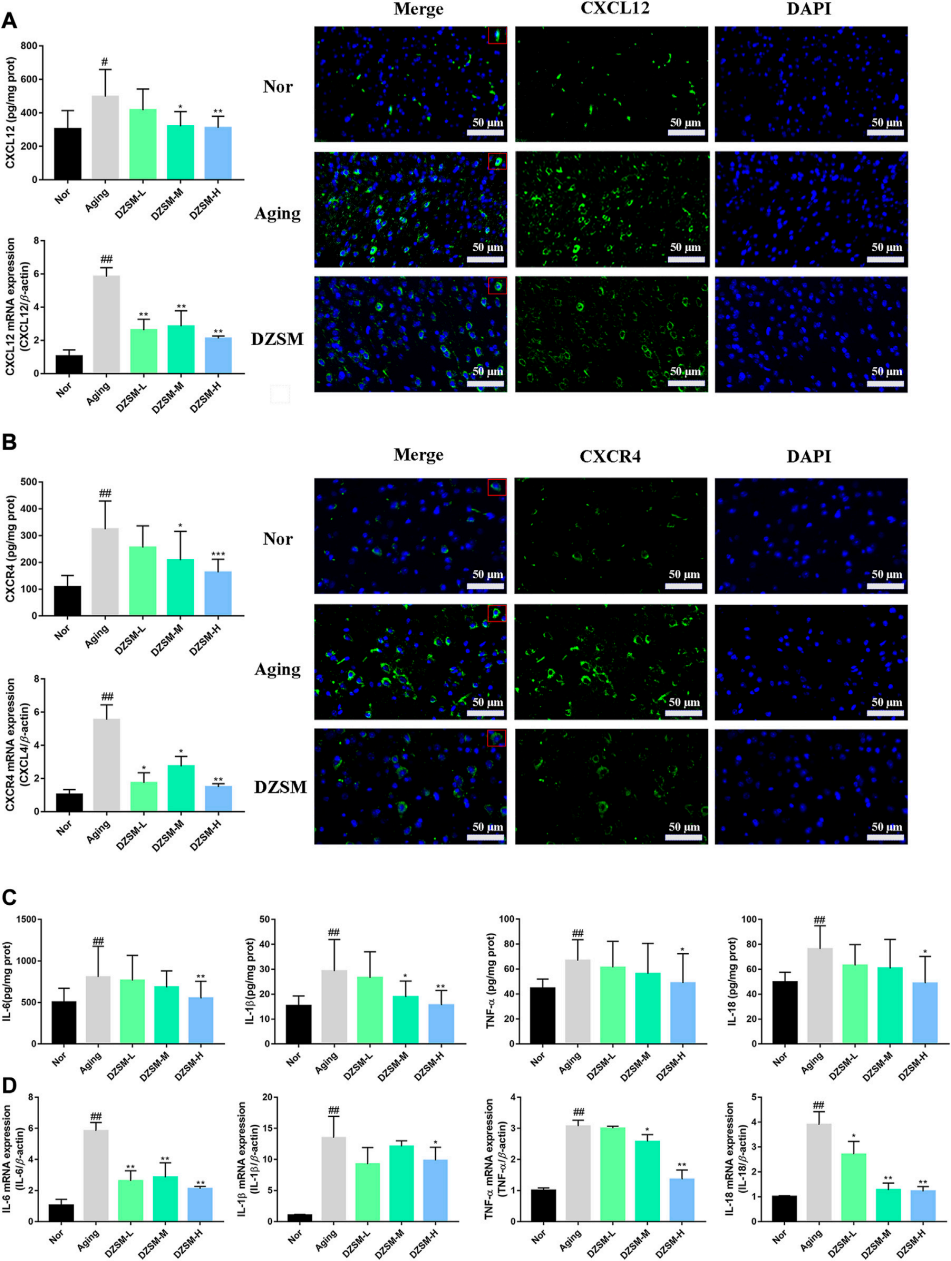
DZSM downregulated CXCR4 and CXCR12 expression and inhibited inflammatory cytokine production. **(A)** RT-PCR, ELISA, and IF analysis of CXCR4 (Bar length: 50 μm); **(B)** RT-PCR, ELISA, and IF analysis of CXCL12 (Bar length: 50 μm); **(C)** Analysis of IL-1β, IL-6, TNF-α, and IL-18 levels by ELISA. **(D)** RT-PCR analysis of IL-1β, IL-6, TNF-α, and IL-18 (compared with the Nor group: ^#^ *p* < 0.05, ^##^ *p* < 0.01; compared with the Aging group: ^*^ *p* < 0.05, ^**^ *p* < 0.01).

CXCL12, also known as stromal cell-derived factor-1 (SDF-1), is the only known ligand of CXCR4, and increasing evidence has indicated that CXCL12/CXCR4 signal transduction participates in regulating neuroimmunology, inflammatory, and neurodegenerative related diseases (Isles et al., 2019; Sanfilippo et al., 2020). As shown in Figures 5A, B, D-galactose led to a sharply increased expression of CXCR4 and CXCL12 at both the protein and gene levels, and these levels were decreased after the DZSM intervention. Recent studies have demonstrated that major pro-inflammatory cytokines, including IL-6, IL-1β, IL-18, and TNF-α, appear to be more closely associated with cognitive dysfunction, and pronounced in elderly and post-operative individuals (Ricker et al., 2011; Hou et al., 2020). Numerous studies have further validated that CXCL12/CXCR4 signal transduction, where directed or undirected, can mediate neuroinflammatory cytokines during cognitive dysfunction, leading to the activation of glial cells and disturbance of immune homeostasis (Shi et al., 2016; Mai et al., 2021). Therefore, our investigation further evaluated the expression of pro-inflammatory cytokines both at the gene and protein levels, as shown in Figures 5C, D, and our results demonstrated that DZSM significantly suppressed the expression of IL-6, IL-1β, IL-18, and TNF-α, compared with that of the Aging group, suggesting that DZSM might work by balancing the immune response through moderating CXCL12/CXCR4 signal transduction and preventing disruption of steady immune homeostasis.

### DZSM effects on intestinal flora

**FIGURE 6:**
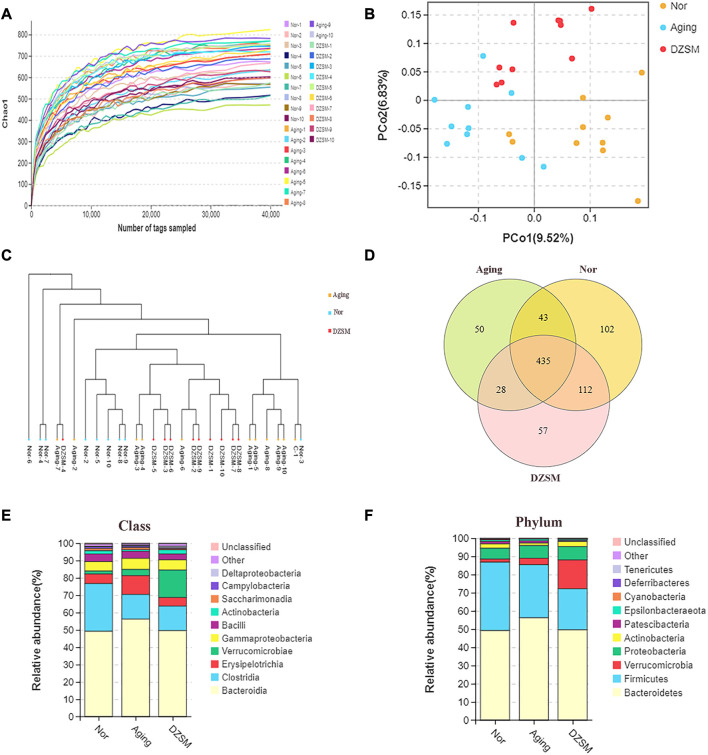
The intestinal microbiome determined by 16S rDNA. **(A)** Rarefaction curve of the chao1 index; **(B)** Unweighted UniFrac PCoA in which each dot represents the gut microbiota of a mouse; **(C)** Unweighted pair group method with arithmetic mean (UPGMA) sample clustering tree; **(D)** Venn diagram of three groups indicates shared and special OTUs of Nor, Aging, and DZSM groups; **(E,F)** Intestinal microbial taxonomic composition of DZSM at the class and phylum levels.

In this study, to investigate the effect of DZSM on mice after D-galactose-induced aging, 30 stool samples were collected, and bacterial 16S rDNA sequencing was performed. The analysis results in a total of 2,804,340 valid reads, which were filtered at a 97% identify threshold to yield 17,790 species-classification OTUs. The chao1 curve (Figure 6A) approached the saturation plateau and suggested that the vast majority of microorganisms were detected by 16S rRNA high-throughput sequencing. As shown in Figures 6B, C, the principal coordinate analysis (PCoA) and unweighted pair group method with arithmetic mean (UPGMA) clustering analysis demonstrated differentials between the Nor, Aging, and DZSM groups. Based on the Venn mapping of Nor vs. Aging vs. DZSM, the diversity of intestinal flora in the Aging group was reduced significantly by 19.12%, compared with the Nor group, and this was reversed in the DZSM intervention group (Figure 6D). As observed in Figure 6E, *Bacteroidia*, *Erysipelotrichia*, *Gammaproteobacteria*, and *Actinobacteria* classes were represented with average loads of 49.18%, 5.54%, 5.41%, and 1.86%, respectively, in the Nor group. In the Aging group, the proportion of Actinobacteria reduced to 0.94%, and the proportion of *Bacteroidia*, *Erysipelotrichia*, and *Gammaproteobacteria* increased sharply to 56.27%, 10.84%, and 6.38%, respectively. In the DZSM group, the levels of *Bacteroidia*, *Erysipelotrichia*, and *Gammaproteobacteria*, reversed to 49.59%, 4.99%, and 5.89%, respectively, and *Actinobacteria* increased to 2.33%. At the phylum level, *Bacteroidetes* accounted for approximately 50%, and DZSM restored the proportion of *Bacteroidetes* induced by D-galactose (56.27%) back to 49.59%. There were also similar tendencies of reversal in the proportions of *Actinobacteria*, *Cyanobacteria*, and *Deferribacteres*, from 2.38%, 0.27%, and 0.19%, respectively, in the Nor group, to 1.32%, 0.18%, and 0.09%, respectively, in the Aging group, and to 2.78%, 0.24%, and 0.11%, respectively, in the DZSM group (Figure 6F).

**FIGURE 7:**
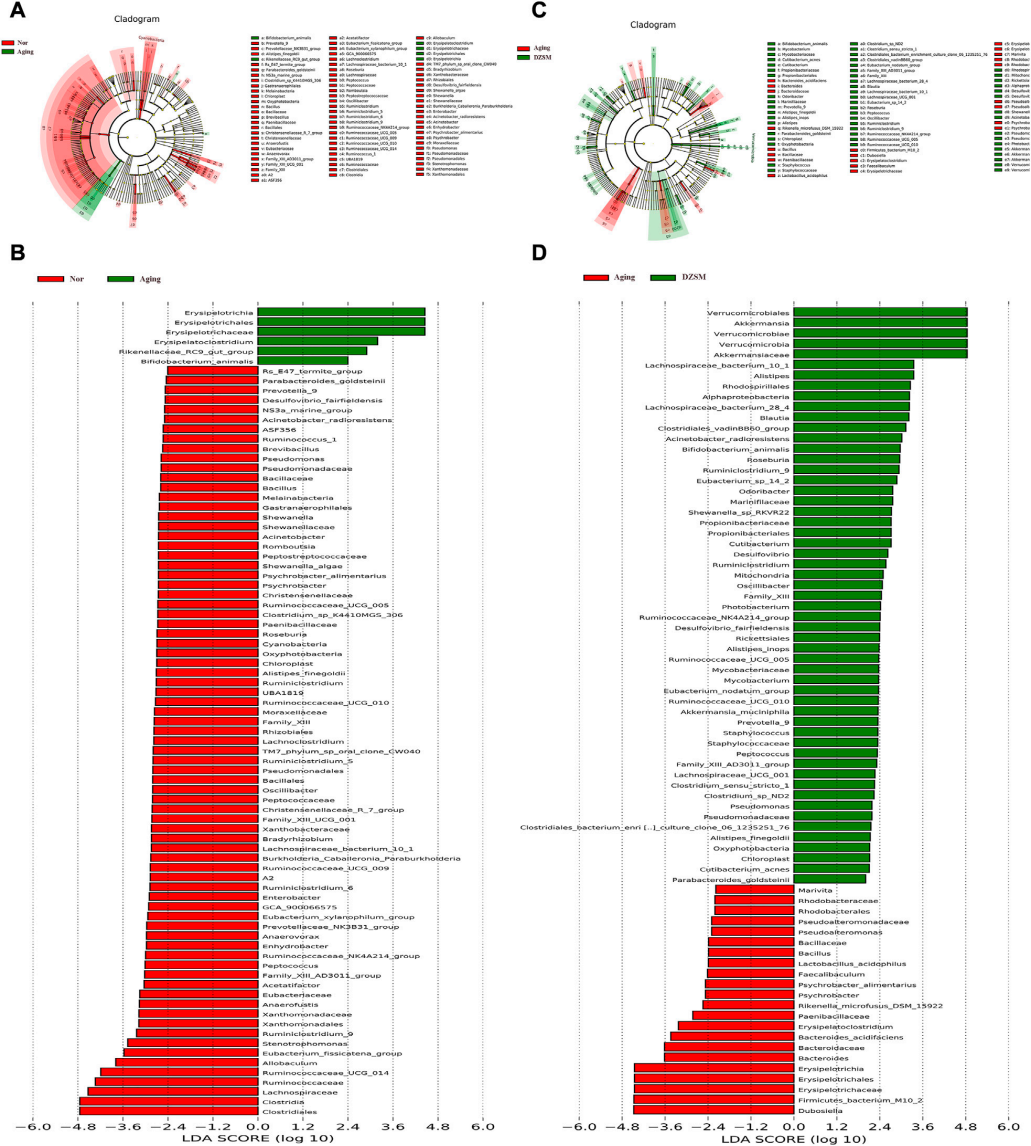
Different species abundance on linear discriminant analysis (LDA) effect size (LefSe). **(A)** Cladogram of Aging vs. Nor groups; **(B)** LDA scores of Aging vs. Nor groups; **(C)** Cladogram of DZSM vs. Aging groups; **(D)** LDA scores of DZSM vs. Aging groups.

To further investigate the alterations of DZSM in aging mice, LEfSe analysis was applied to identify and compare the key taxa contributing to the different microbiota compositions of the Nor, Aging, and DZSM-H groups. As shown in Figures 7A, B six biomarkers, including *Erysipelotrichia*, *Erysipelotrichales*, *Erysipelotrichaceae*, *Erysipelatoclostridium*, *Rikenellaceae_RC9_gut_group*, and *Bifidobacterium_animalis*, were enriched in the Aging group. Furthermore, 20 taxa (*Prevotella_9*, *Alistipes_finegoldii*, *Parabacteroides_goldsteinii*, *Oxyphotobacteria*, *Chloroplast*, *Family_XIII*, *Family_XIII_AD3011_group*, *Lachnospiraceae_bacterium_10_1*, *Roseburia*, *Peptococcus*, *Oscillibacter*, *Ruminiclostridium*, *Ruminiclostridium_9*, *Ruminococcaceae_NK4A214_group*, *Ruminococcaceae_UCG_005*, *Ruminococcaceae_UCG_010*, *Desulfovibrio_fairfieldensis*, *Acinetobacter_radioresistens*, *Pseudomonadaceae*, and *Pseudomonas*) were more abundant in the DZSM group and overlapped with the Nor group (Figures 7C, D).

### DZSM regulated CXCL12/CXCR4 signal transduction and inhibited inflammatory cytokines, improving microbes according to integrated transcriptomics and intestinal microbiota analysis

**FIGURE 8:**
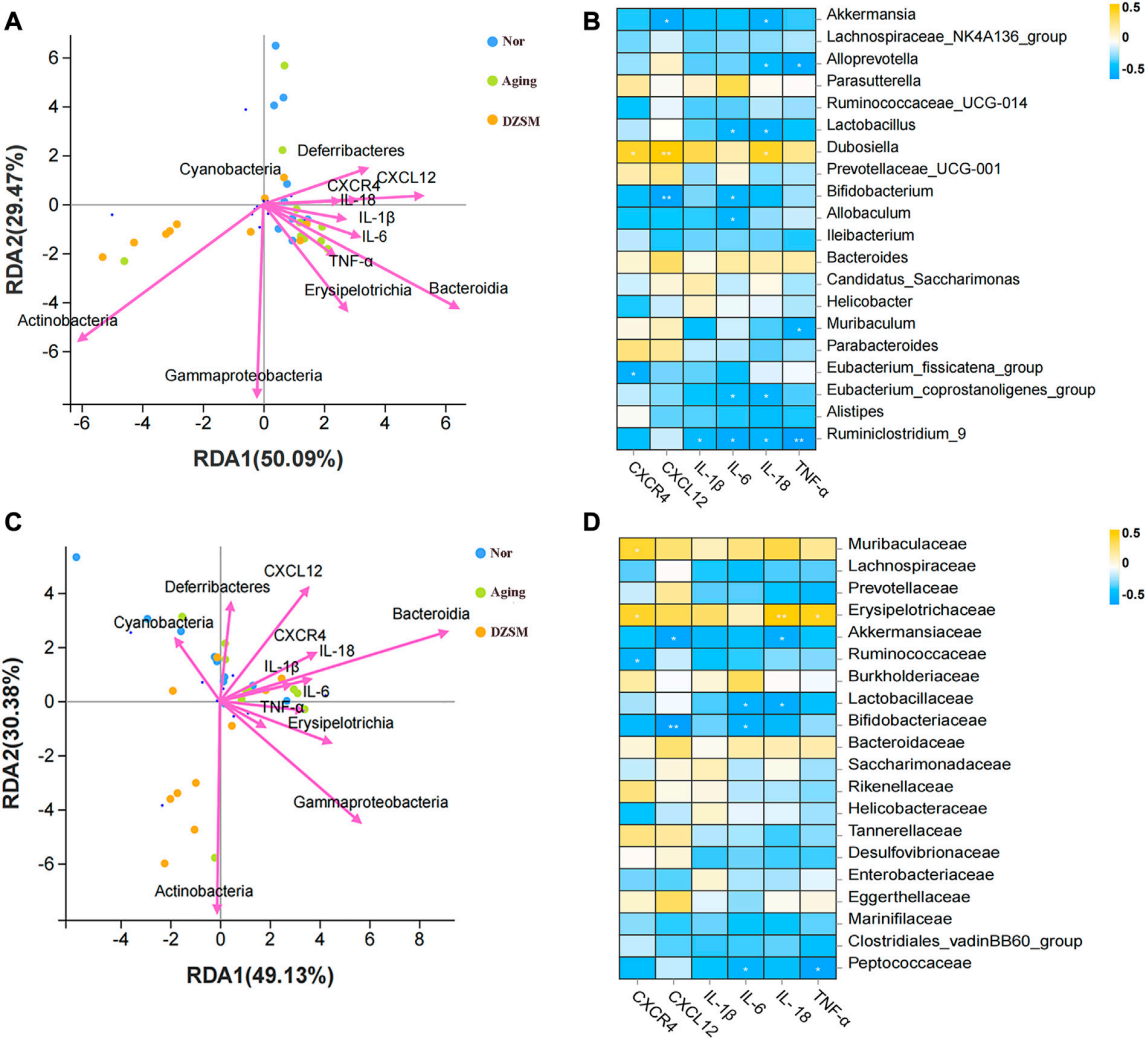
Relationship between the relative abundance of gut microbiota and key targets and pro-inflammatory cytokines. **(A)** Redundancy analysis (RDA) between microbes of the genus and key targets and pro-inflammatory cytokines; **(B)** Correlations between the microbes of the main genus and the key targets and pro-inflammatory cytokines; **(C)** Redundancy analysis (RDA) between the microbes of the family and key targets and pro-inflammatory cytokines; **(D)** Correlations between the microbes of the main family and the key targets and pro-inflammatory cytokines.

The relationship between the gut microbiota and the key targets/pro-inflammatory cytokines was investigated using RDA and Spearman correlation analyses. As Figures 8A, C demonstrate, *Bacteroidia, Erysipelotrichia,* and *Deferribacteres* showed close positive associations with CXCR4, CXCL12, and inflammatory factors IL-1β, IL-6, IL-18, and TNF-α, both at genus and family levels. *Akkermansia*, *Bifidobacterium*, and *Ruminiclostridium_9* were negatively correlated with CXCR4 and CXCL12, as well as inflammatory factors IL-1β, IL-6, IL-18, and TNF-α at the genus level. *Akkermansiaceae*, *Lactobacillaceae*, *Bifidobacteriaceae*, and *Peptococcaceae* were also negatively associated with CXCR4, CXCL12, IL-1β, IL-6, IL-18, and TNF-α at the family level (Figures 8B, D). These results suggested that the DZSM exerted ameliorating CI effects in D-galactose-induced aging mice *via* regulation of the distribution and composition of the gut microbiota and the release of inflammatory factors.

## Discussion

The aging of the global population is a rapidly growing phenomenon, which not only triggers many age-related diseases but also imposes significant social and economic burdens on society. One of the well-known features of the aging process is the development of cognitive deficits and impairments in learning and memory (Go et al., 2018; Khan et al., 2019). The present study investigated the neuroprotective effects of DZSM, a traditional Chinese formulation, against CI in D-galactose-induced aging mice. Using an accelerating aging mouse model, DZSM (0.162 g/kg/d or 0.486 g/kg/d) treatment for 6 weeks was found to improve learning and memory abilities, reduce neural loss, and repair Nissl body morphology. Transcriptomics and gut microbiota analysis suggested that DZSM’s therapeutic effect may be mediated through CXCR4 by regulating inflammatory responses and signal transduction processes, while *Bacteroidia*, *Erysipelotrichia*, and *Deferribacteres* may be key in alleviating CIs. Additionally, DZSM was found to decrease the levels of CXCR4, CXCL12, and multiple pro-inflammatory cytokines, including TNF-α, IL-6, IL-1β, and IL-18 in aging mice, indicating its ability to mitigate neuroinflammation and exert a neuroprotective effect.

Chronic exposure to D-galactose (i.p.) is a reliable method for inducing aging and CI models, as it triggers multiple symptoms such as neuroinflammation, oxidative stress, neurodegeneration, and learning and memory impairments (Li et al., 2019). To replicate the complex pathological features of aging, we established an aging-like mouse model using D-galactose (i.p.) in this study. Our findings indicated that the D-galactose-induced mice exhibited significant cognitive dysfunction and pathological impairment, consistent with previous studies, including increased neuroinflammation, oxidative damage, and decreased exploration, learning, and memory abilities (Budni et al., 2016; Sun et al., 2018). Interestingly, DZSM, with its various anti-oxidative, anti-inflammatory, and neuroprotective agents such as scutellarin, caffeic acid, ginsenoside Rb3, ginsenoside Rg1, ginsenoside Re, and schisandrin, may function as a crucial mediator of cognitive deficits (Chen et al., 2016; Zhang et al., 2020). Furthermore, the herbs that compose DZSM, including *E. breviscapus* (Vaniot) Hand.-Mazz.*, P. ginseng* C.A. Mey., *O. japonicus* (Thunb.) Ker-Gawl., and *S. chinensis* (Turcz.) Baill., have shown multiple pharmacological activities in both experimental and clinical applications (Lin et al., 2019; Wan et al., 2021). Hence, these bioactive compounds and herbs may form the basis for the beneficial role of DZSM in protecting against cognitive dysfunction.

To perform high-throughput characterization of the gene expression profiles at the tissue level, RNA-Seq was conducted on brain tissue. Transcriptomic analysis revealed that 196 of DEGs were reversed in the Aging-vs.-Nor and DZSM-vs.-Aging groups, indicating that the mechanism of DZSM in improving CI is polygenic, rather than involving a single gene. To identify key targets, for the combined treatment of cognitive deficits with DZSM, a PPI network was constructed. CXCR4 (degree = 19), a member of a family of highly conversed G protein-coupled receptors, was identified as a key target. CXCR4 has modulatory properties of neuron-glial interactions in the peripheral or central nervous system, such as in the neurons, astrocytes, and microglia (Li and Ransohoff, 2008). Recent studies indicated its vital role in neuromodulation, neuroprotection, and neuron-glial interaction communication, even improving neurological disorders such as infections, ischemia, cancer, and multiple unknown injuries (Hsiao et al., 2021). CXCL12, the ligand of CXCR4, regulates neuronal firing and neuron-glial interactive communication (Li et al., 2018). To date, increasing evidence has indicated that CXCL12 binding to CXCR4 triggers signal transduction pathways, showing multiple inflammatory-modulating activities, including recruiting of perivascular macrophages in neuropathy (Mai et al., 2021), activating cells to secrete and release inflammatory cytokines (Dong et al., 2021), and promoting neuronal circuit formation (Peng et al., 2007). The results of quantitative real-time PCR and immunostaining shown that the expression of CXCL12 and CXCR4 was significantly suppressed in a dose-dependent manner after treatment with DZSM, indicating that DZSM exhibited neurocognitive function effects by modulating CXCR4 and CXCL12. Growing interest in the complex network of inflammatory mediators and immune system has enabled the identification of a growing number of pro-inflammatory molecules associated with CI and aging, such as IL-6, TNF-α, IL-18, and IL-1β (Cattaneo et al., 2017). Interestingly, chronic administration of DZSM was found to suppress neuroinflammation and alleviate the effects of aging and CI, as evidenced by the decreased levels of pro-inflammatory cytokines IL-6, TNF-α, IL-18, and IL-1β in aging mice after DZSM administration. These results suggest that DZSM could be a potential therapeutic agent for CI by suppressing neuroinflammation and modulating CXCR4 and CXCL12.

**FIGURE 9:**
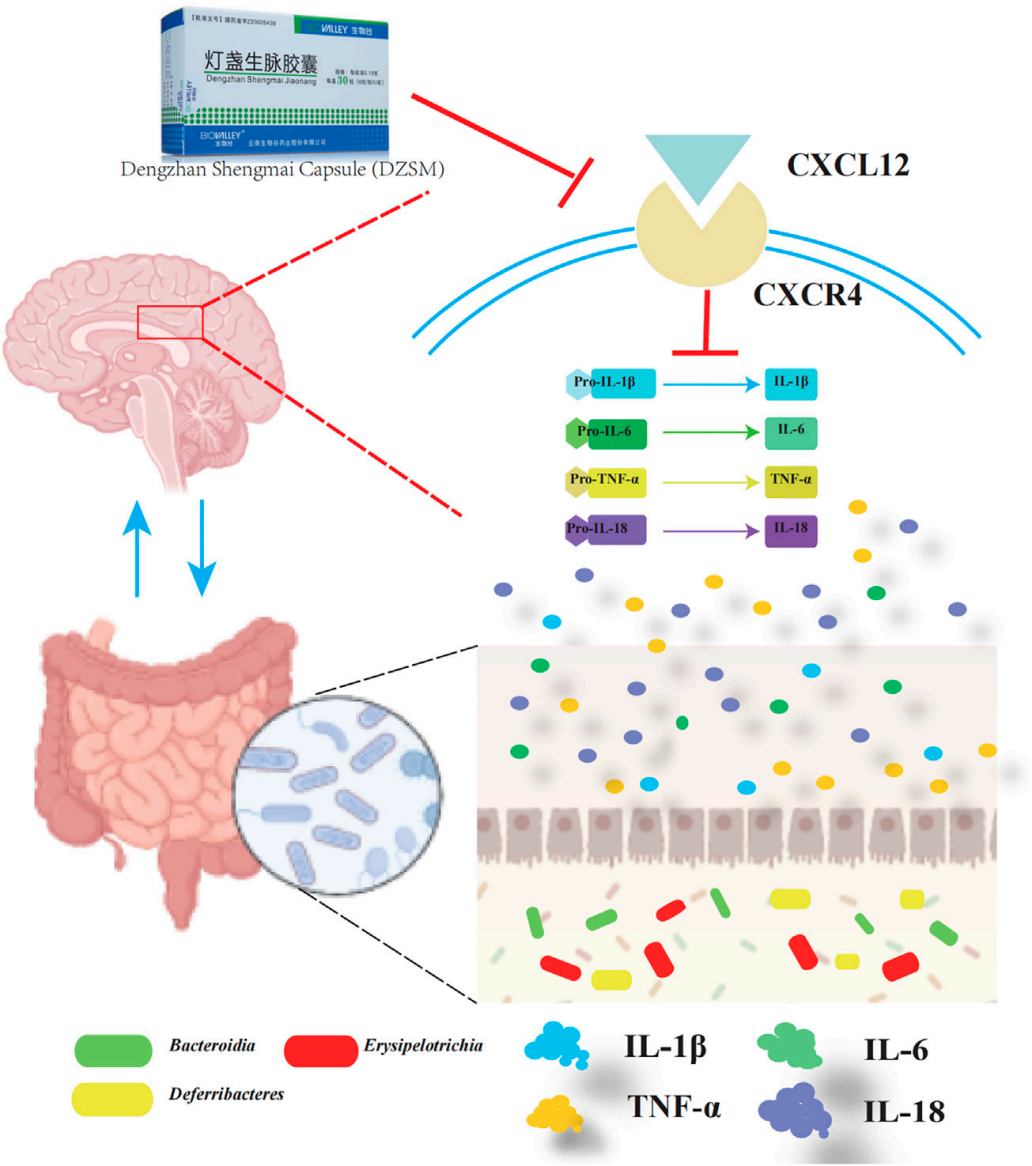
Overview of the cognitive impairment-ameliorating mechanism of DZSM *via* CXCL12/CXCR4 regulation and microbial improvement.

Multiple studies have shown that gut microbiota colonization is strongly associated with CI due to its adverse effects on metabolic disturbances or the progression of low-grade inflammation leading to neurological dysfunction. Our findings are consistent with previous research indicating that a shift in the proportion of *Bacteroidia*, *Erysipelotrichia*, and *Deferribacteres* is closely related to increased inflammation (Jiao et al., 2018; Ren et al., 2021). Furthermore, our comprehensive analysis integrating transcriptomics and gut microbiota indicates that CXCL12 and CXCR4 modulate pro-inflammatory factors like IL-1β, IL-6, IL-18, and TNF-α and indirectly influence the composition or distribution of *Bacteroidia*, *Erysipelotrichia*, and *Deferribacteres* (Ng et al., 2011) (Figure 9). Our results suggest that the DZSM group had higher levels of *Deferribacteres* and lower levels of *Bacteroidia* and *Erysipelotrichia* compared to the Aging group. These changes were positively correlated with CXCR4, CXCL12, and inflammatory factors such as IL-1β, IL-6, IL-18, and TNF-α. We propose that the therapeutic benefits of DZSM for CI may be linked to its ability to regulate the inflammatory response and modulate the intestinal flora. Furthermore, our results suggest that the interaction between inflammatory cytokines and intestinal flora may contribute to the age-delaying effects of DZSM. Notably, *Bacteroidetes*, which constitute a significant proportion of the gut microbiota, have been shown to confer beneficial effects. Recent research indicates that the phylogenetic composition of human and mouse bacterial communities is similar at the phylum level, with *Bacteroidetes* and *Firmicutes* being the dominant phyla (Ley et al., 2006; Rawls et al., 2006). Coelho et al. (2018) have demonstrated that the gene content of *Bacteroidetes* and its response to diet are similar in both species. These findings suggest that the discovery of intestinal flora in mouse models may serve as a predictor of human microbiome results candidate biomarkers for early diagnosis (Kim et al., 2021). Our study also found a positive correlation between the proportion of *Bacteroidetes* and neurocognitive function, which is consistent with prior research (Shi et al., 2020; Xie et al., 2020). Meanwhile, in human microbiome studies, alterations in the proportion of *Bacteroidetes* have been linked to cognitive function. For instance, infants with higher *Bacteroidetes* strains at 1 year of age exhibit better cognitive performance at 2 years of age (Mariat et al., 2009), whereas cognitively impaired patients have lower abundances of *Bacteroidetes* strains in their gut microbiota (Carlson et al., 2018). *Erysipelotrichia,* a class of *Firmicutes* (Bhattacharjee et al., 2022), has also been found to be similar in both human and mouse flora (Coelho et al., 2018). Our study revealed a close association between the proportion of *Erysipelotrichia* and inflammatory cytokines, such as IL-6, IL-1, IL-1β, and IL-18 (Tang et al., 2022). *Erysipelotrichia* has been reported to produce high levels of anti-inflammatory short-chain fatty acids (Zhu et al., 2022) and is involved in immune regulation. However, it is also closely related to the production of inflammatory factors (Pindjakova et al., 2017), such as IL-6, IL-1 IL-1β, and IL-17, and has been found to be closely related to inflammatory diseases by Griffen et al. (2012). They performed the 16S pyrophosphate to detect the flora of healthy control and patients, further confirming that *Erysipelotrichia* was closely related to the prevalence of inflammatory diseases. *Deferribacteres*, on the other hand, is a pro-inflammatory bacterium that exhibits a protective effect by reducing low-grade chronic inflammation. It is closely associated with the pro-inflammatory cytokines IL-6, TNF-α, and IL-17A (Li et al., 2019), which affect the cognitive function of the brain and the whole organism (Liang et al., 2018). In addition, alterations in the proportion of *Deferribacteres* have been also considered as potential microbiome markers for early diagnosis in clinical application. For instance, Kim et al. (2021) identified *Deferribacteres* as a candidate biomarker for early diagnosis of pancreatic cancer using microbial extracellular vesicles to detect microbiome compositional differences in blood samples from patients with pancreatic cancer and healthy controls. Although our study elucidated the mechanisms of DZSM in improving CIs, it remains unclear which of the main ingredients act individually or synergistically to create the observed effects. Therefore, further clinical and basic experiments are needed to investigate and identify the fundamental ingredients of DZSM in improving CIs.

## Conclusion

The current study provides evidence that DZSM supplementation improved memory and cognitive function in a D-galactose-induced aging mouse model *via* comprehensively integrated transcriptomics and gut microbiota analyses. The underlying mechanism, whereby DZSM exerts its ameliorating effect on cognitive deficits appears to be mediated by the regulation of CXCL12/CXCR4, suppression of the expression of inflammatory cytokines, and adjustment of the intestinal flora composition, including *Bacteroidia*, *Erysipelotrichia*, and *Deferribacteres* species.

## Data Availability

The datasets presented in this study can be found in online repositories. The names of the repository/repositories and accession number(s) can be found below: NCBI BioProject (https://www.ncbi.nlm.nih.gov/bioproject/), PRJNA944587 and PRJNA946342.

## Supplementary material

SUPPLEMENTARY FIGURE S1 UPLC/MS analysis of DZMS. **(A)** Chromatogram of DZSM in positive ESI mode; **(B)** Chromatogram of DZSM in negative ESI mode.

## Glossary

### The following abbreviations are used in this document:

AD: Alzheimer’s Disease
ANOVA: Analysis of variance
BP: Biological process
BSA: Bovine Serum Albumin
CC: Cellular component
CI: Cognitive impairment
CXCL12: CXC chemokine ligand 12
CXCR4: Chemokine CXC motif receptor 4
DEG: Differentially expressed genes
DZSM: Dengzhan Shengmai
ELISA: Enzyme-linked immunosorbent assay
ESI: electrospray ionization
GO: Gene Ontology
H&E: Hematoxylin and eosin
HRP: Horseradish peroxidase
IL-1β: Interleukin-1β
IL-6: Interleukin-6
IL-18: Interleukin-18
KEGG: Kyoto Encyclopedia of Genes and Genomes
LDA: Linear discriminant analysis
MF: Molecular function
MMSE: Mini-mental State Examination
MoCA: Montreal Cognitive Assessment
MWM: Morris water maze
OFT: Open field task
OTU: Operational taxonomic units
PBS: Phosphate-buffered saline
PCoA: principal coordinates analysis
PCR: Polymerase chain reaction
PPI: Protein–protein interaction
RDA: redundancy analysis
RNA: Ribonucleic acid
SCFAs: short-chain fatty acids
TCM: Traditional Chinese medicine
TIC: total ion chromatogram
TNF-α: Tumor necrosis factor-α
UPGMA: Unweighted pair group method with arithmetic.
