# Supplementary material for: Multi-omics reveals Dengzhan Shengmai formulation ameliorates cognitive impairments in D-galactose-induced aging mouse model by regulating CXCL12/CXCR4 and gut microbiota
Source: Front Pharmacol. 2023 Apr 10;14:1175970. doi: 10.3389/fphar.2023.1175970 (PMC10123283; doi:10.3389/fphar.2023.1175970)
Supplement: Supplementary file 1 [file Table1.DOCX]

**Table 1 Primers for RT-qPCR**

| **NO.** | **Gene** | **Sequence (5’-3’)** |
| --- | --- | --- |
| 1 | CXCR4 | Forward: AAAGCTAGCCGTGATCCTCA |
|  |  | Reverse: CACCATTTCAGGCTTTGGTT |
| 2 | CXCL12 | Forward: CTTCATCCCCATTCTCCTCA |
|  |  | Reverse: GACTCTGCTCTGGTGGAAGG |
| 3 | TNF-α | Forward: TAGCCAGGAGGGAGAACAGA |
|  |  | Reverse: TTTTCTGGAGGGAGATGTGG |
| 4 | IL-6 | Forward: TCCAGTTGCCTTCTTGGGAC |
|  |  | Reverse: GTGTAATTAAGCCTCCGACTTG |
| 5 | IL-1β | Forward: GACCTTCCAGGATGAGGACA |
|  |  | Reverse: AGCTCATATGGGTCCGACAG |
| 6 | IL-18 | Forward: ACGTGTTCCAGGACACAACA |
|  |  | Reverse: ACAAACCCTCCCCACCTAAC |
| 7 | β-actin | Forward: TGTTACCAACTGGGACGACA |
|  |  | Reverse: GGGGTGTTGAAGGTCTCAAA |
