# Supplementary figures and images for: Multi-omics reveals Dengzhan Shengmai formulation ameliorates cognitive impairments in D-galactose-induced aging mouse model by regulating CXCL12/CXCR4 and gut microbiota
Source: Front Pharmacol. 2023 Apr 10;14:1175970. doi: 10.3389/fphar.2023.1175970 (PMC10123283; doi:10.3389/fphar.2023.1175970)

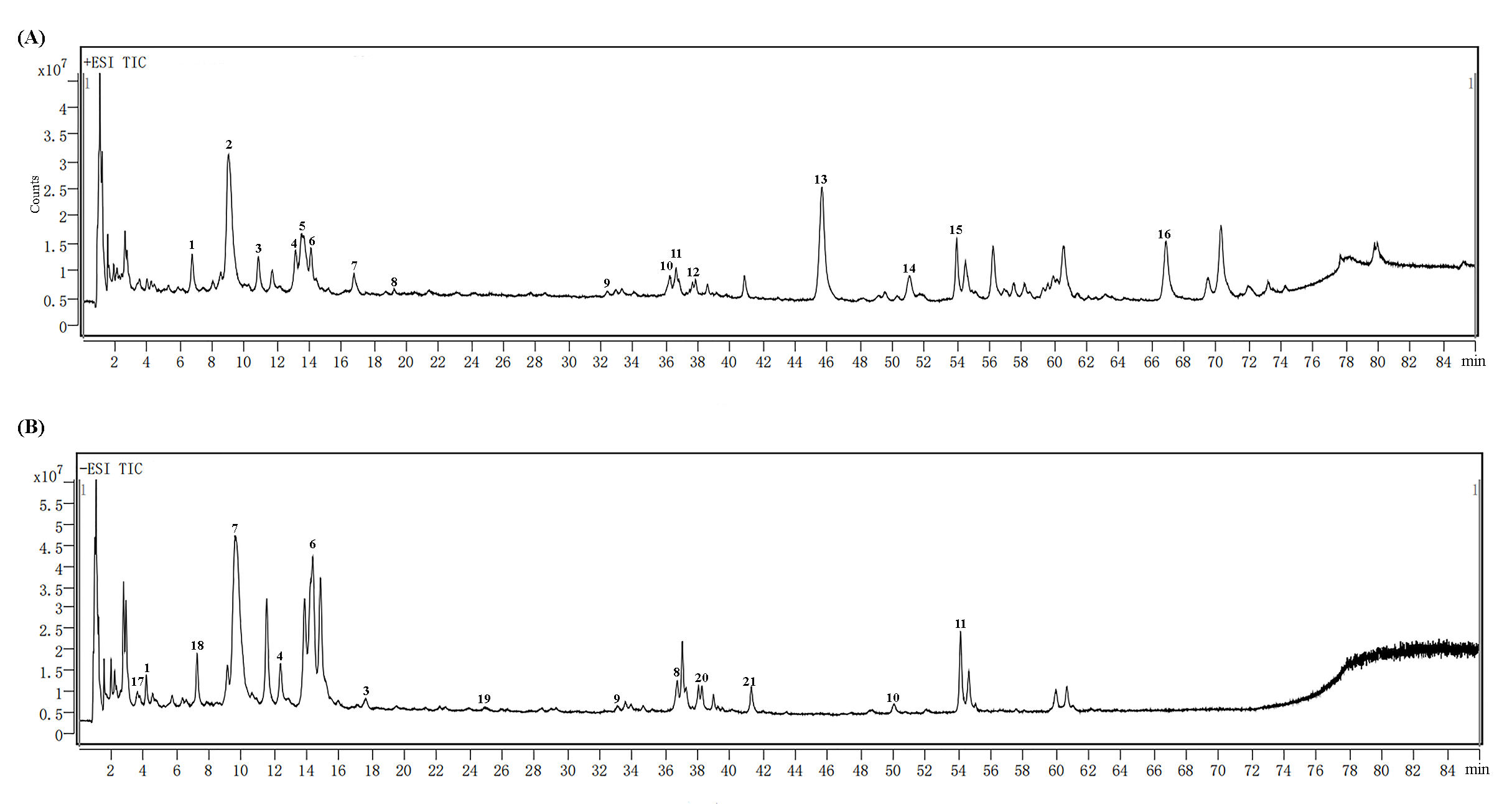

Supplement: Supplementary file 2 [file Image1.TIF]
